# HDAC4 and 5 repression of TBX5 is relieved by protein kinase D1

**DOI:** 10.1038/s41598-019-54312-w

**Published:** 2019-11-29

**Authors:** Tushar K. Ghosh, José J. Aparicio-Sánchez, Sarah Buxton, J. David Brook

**Affiliations:** School of Life Sciences, Queen’s Medical Centre, University of Nottingham, Nottingham, NG7 2UH UK

**Keywords:** Biochemistry, Development

## Abstract

TBX5 is a T-box family transcription factor that regulates heart and forelimb development in vertebrates and functional deficiencies in this protein result in Holt-Oram syndrome. Recently, we have shown that acetylation of TBX5 potentiates its activity and is important for heart and limb development. Here we report that class II histone deacetylases HDAC4 and HDAC5 associate with TBX5 and repress its role in cardiac gene transcription. Both HDAC4 and HDAC5 deacetylate TBX5, which promotes its relocation to the cytoplasm and HDAC4 antagonizes the physical association and functional cooperation between TBX5 and MEF2C. We also show that protein kinase D1 (PRKD1) relieves the HDAC4/5-mediated repression of TBX5. Thus, this study reveals a novel interaction of HDAC4/5 and PRKD1 in the regulation of TBX5 transcriptional activity.

## Introduction

Histone deacetylases (HDACs) are histone modifying enzymes that remove the acetyl group from histone and non-histone proteins and control vital cellular processes such as cell proliferation, differentiation and development^1,2^. Based on their structure and function they are classified into four different groups: class I (HDACs 1, 2, 3 and 8), class II (class IIa - HDACs 4, 5, 7, 9 and class IIb – HDACs 6 and 10), class III (the sirtuins, SIRT 1–7) and class IV (HDAC11)^3^.

The class I HDACs are highly similar to yeast RPD 3 (reduced potassium dependency 3) and are ubiquitously expressed^4^. HDAC1 and HDAC2 share 85% similarity and play a redundant role in cardiac morphogenesis^5^. Deletion of *Hdac1* in mice produces embryonic lethality due to proliferation defects^6^. *Hdac2* null mice were unable to reactivate the fetal gene program or failed to exhibit normal hypertrophic responses. In addition, transgenic overexpression of *Hdac2* in the heart induces cardiac hypertrophy^7^. HDAC3 is predominantly located in the nucleus and it shows less similarity to HDAC1 and 2 than they do to each other. Cardiac specific deletion of *Hdac3* in mice produces massive cardiac hypertrophy and upregulation of genes associated with fatty acid metabolism^8^. In addition, mice overexpressing *Hdac3* in the heart show a distinct proliferation of postnatal cardiac myocytes, but without hypertrophy^9^.

Class II HDACs are highly similar to yeast HDA1 (histone deacetylase-A 1) and suppress heart growth^10^. They are abundantly expressed in the heart, brain and skeletal muscles^11^ and are signal responsive repressors of cardiac hypertrophy^10^. Calcium/calmodulin-dependent protein kinase II (CaMKII) phosphorylates HDAC4, promoting its nuclear export and de-repression of myocyte enhancer factor-2 (MEF2) target genes in cardiomyocytes^12,13^. Protein kinase CaMKII specifically transmits signals via a unique docking site in HDAC4 that is absent from other class IIa HDACs^12^. HDAC5 acquires CaMKII responsiveness by interacting with HDAC4^14^. Protein kinase C and D mediate agonist-dependent cardiac hypertrophy through nuclear export of HDAC5^15^ and in H9C2 cells, CaMKIV and protein kinase D regulate nucleocytoplasmic localization of HDAC5^16^.

Acetylation potentiates the transcriptional activity of TBX5^17^ and histone deacetylases are key players in heart development and cardiac hypertrophy^10^. Here we investigated the possible link between histone deacetylases and TBX5. We report that TBX5 associates with both class I and class II HDACs and the association of class II HDACs with TBX5 results in suppression of cardiac gene transcription. HDAC4 competes with and disrupts the functional cooperation between TBX5 and MEF2C, which plays a key role in early heart development. HDAC4/5-mediated gene repression can be partially rescued by Protein kinase D1 (PRKD1). These studies suggest the TBX5-mediated gene regulatory pathway is linked to a signal-mediated protein kinase via PRKD1.

## Results

### Physical interaction of TBX5 with HDACs and functional consequences

**Figure 1:**
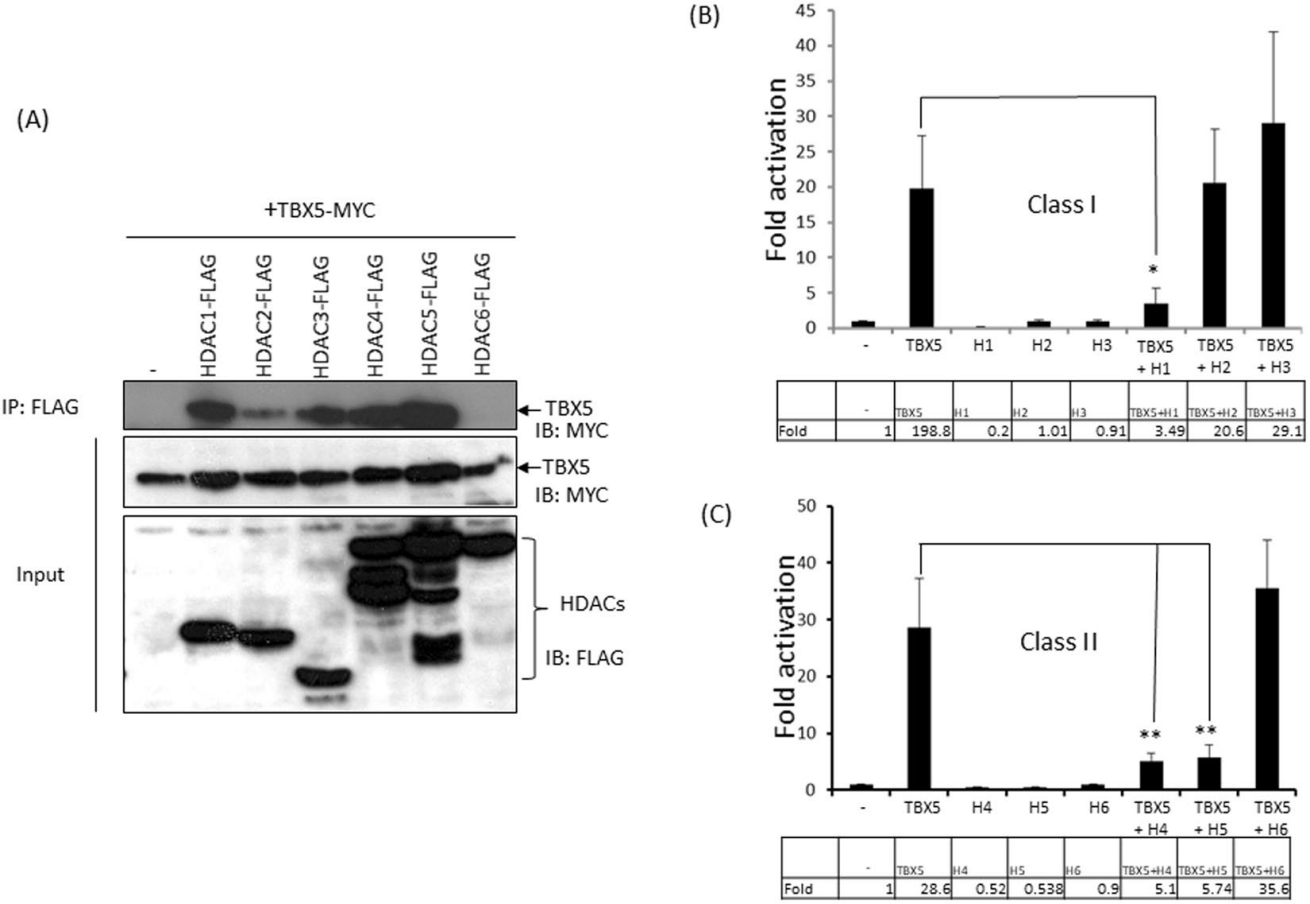
Physical interaction of TBX5 with HDACs and functional consequences. (**A**) Pull-down assay showing the physical interaction between TBX5 and class I and class II HDACs. HDACs (1–5) interact with TBX5, whereas HDAC6 does not (IP: immunoprecipitation and IB: Immunoblot). Full-length blots are shown in Supplementary Fig. S1. (**B**,**C**) Luciferase-reporter assays showing that class I histone deacetylase HDAC1 and class II histone deacetylases HDAC4 and 5 repress TBX5-mediated transcriptional activity on the MYH6 promoter. Results are from three individual experiments. Error bars represent SD, *P < 0.02, **P < 0.01. Inset showing the Fold changes.

Histone acetyltransferases KAT2A/2B acetylate TBX5^17^. Histone deacetylases (HDACs) are transcriptional repressors and anti-hypertrophic. Given the counteracting role of HDACs in gene regulation, we investigated whether HDACs interact with TBX5 and modulate transcription. To analyse their physical interaction, we co-transfected Cos7 cells with plasmids encoding MYC-TBX5 along with each of six plasmids encoding FLAG-HDACs (1–6). Pull-down and Western blot analyses were carried out on cell extracts to examine their physical interaction. Figure 1A shows that class I HDACs (1–3) and class II HDACs (4 and 5) interact with TBX5. Whereas TBX5 strongly interacts with HDAC 1, 3, 4 and 5, a relatively weak interaction was observed with HDAC2, and HDAC6 did not interact with TBX5.

In light of this interaction we sought to examine the effect of both class I and class II HDACs on TBX5-mediated gene transcription. We performed reporter assays on pGL3-*MYH6*, a luciferase reporter gene construct under the control of the TBX5 target promoter *MYH6*. Reporter assays were performed in cardiomyoblast cells H9c2. As shown in Fig. 1B, HDAC1 produced a strong repression on basal promoter activity of *MYH6*, whereas HDAC2 and HDAC3 did not. We then tested the effect of HDACs on TBX5-mediated transcription and found that HDAC1 led to a 75% reduction of the TBX5-mediated activation on this promoter (P < 0.02). HDAC2 did not show any effect, whereas HDAC3 increased the TBX5-mediated activation, although it was not statistically significant (P > 0.05).

Class II histone deacetylases HDAC4 and 5 repressed the basal promoter activity of *MYH6* by almost 50%, whereas HDAC6 had no effect (Fig. 1C). When either HDAC4 or HDAC5 was co-transfected with TBX5 they strongly inhibited the TBX5-mediated activation (80% reduction, P < 0.01). Co-transfection of HDAC6 with TBX5 enhanced the TBX5-mediated activation, but this increase was not statistically significant (P > 0.05).

Overall, these experiments suggest that HDAC1, 4 and 5 repress TBX5-mediated transcription, whereas HDAC3 and 6 did not have a significant effect on transcription from the *MYH6* promoter.

### HDAC4 and HDAC5 deacetylate TBX5

**Figure 2:**
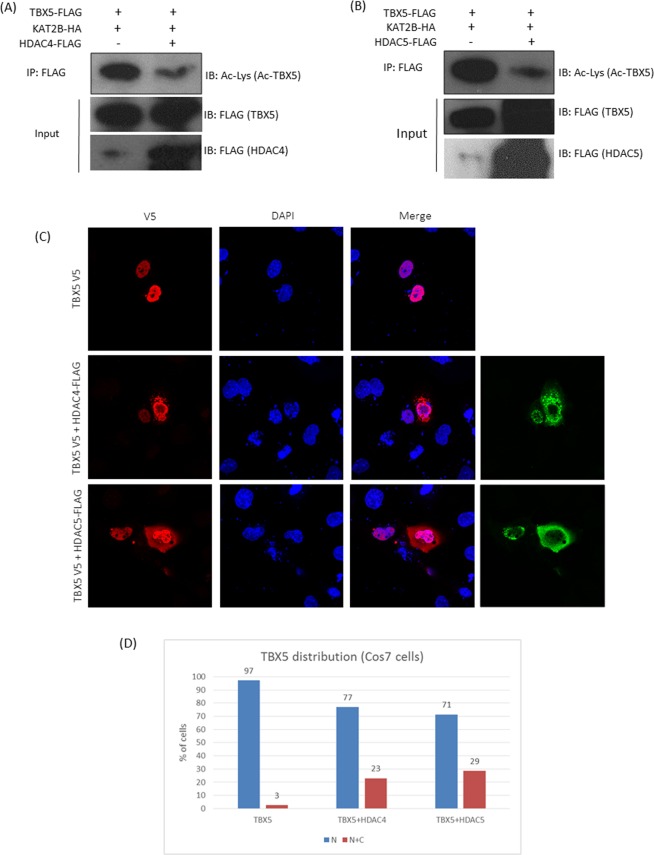
HDAC4 and HDAC5 deacetylate TBX5 and promote its nuclear export. (**A**,**B**) Pull-down and Western blot analysis showing that both HDAC4 and HDAC5 strongly deacetylate TBX5. KAT2B was used to promote TBX5 acetylation. Full-length blots are shown in Supplementary Fig. S2 (**A**–**D**). (**C**) Representative images of the cellular distribution of TBX5 following HDAC4/HDAC5 overexpression, showing the partial re-localization of TBX5 into the cytoplasm. (**D**) Cell count showing the cellular distribution of TBX5 in cells transfected with TBX5 alone or TBX5 and HDAC4/5 (TBX5- total counts 300; 292 (N) and 8 (N + C), TBX5 + HDAC4- total cell counts 257; 198(N) and 59 (N + C) and TBX5 + HDAC5− total counts 274; 195(N) and 79(N + C). Results are from three individual experiments (N = nuclear, C = cytoplasmic).

Acetyltransferases KAT2A/2B acetylate TBX5 and enhance transcriptional activity by promoting its nuclear retention^17^. Since HDAC4 and 5 interact with TBX5 and repress TBX5-mediated transcription, we investigated whether the reduced transcriptional activity could be partly due to deacetylation of TBX5 and its subsequent nuclear export. To assess HDAC4/5 deacetylase activity on TBX5, Cos7 cells were co-transfected with the expression plasmids for TBX5-FLAG and KAT2B-HA along with either FLAG-HDAC4 or FLAG-HDAC5. The FLAG-TBX5 protein was affinity purified on Anti-FLAG-agarose beads from the cell extracts and the acetylation level of TBX5 was monitored by Western blot analysis using anti-acetylated lysine antibody. As shown in Fig. 2A,B, overexpression of HDAC4 or HDAC5 significantly reduced the acetylation level of TBX5, suggesting a role for these two HDACs in TBX5 deacetylation.

In order to investigate whether TBX5 deacetylation has an effect on TBX5 cellular localization, we performed immunofluorescence staining on Cos7 cells co-transfected with TBX5-V5 and either of HDAC4-FLAG or HDAC5-FLAG. As expected, TBX5-V5 alone is exclusively nuclear in 100% of the cells we checked. However, in the presence of HDAC4 or HDAC5 we found that TBX5-V5 was also present in the cytoplasm in 20–30% of cells, indicating that the overexpression of class II HDACs relocate a certain amount of TBX5 protein to the cytoplasm (Fig. 2C,D).

### HDAC4/5 inhibit the transcriptional activity of TBX5-K339R

**Figure 3:**
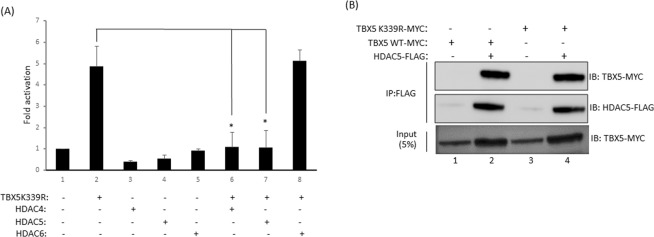
HDAC4 and HDAC5 repress TBX5K339R-mediated transcription. (**A**) Promoter-reporter assay showing that HDAC4 and 5 can repress the transcriptional activity of TBX5 mutant K339R. HDAC6, which does not interact with TBX5, failed to repress the function of TBX5-K339R. Values are mean ± SD (n = 3), *P < 0.001. (**B**) Pull-down assay shows that TBX5-K339R retains the binding activity to HDAC5. Full-length blots are shown in Supplementary Fig. S3 (**A**–**C**).

Acetylation modulates TBX5 activity by regulating its nucleocytoplasmic distribution. HDAC4 and 5 deacetylate and partially relocate TBX5 into the cytoplasm thereby reducing TBX5 mediated gene transcription. However, HDAC4 and 5 can also regulate gene transcription by chromatin remodelling via promoter recruitment. To understand whether chromatin modification is part of the TBX5 regulatory mechanism, we performed reporter assays using the acetyl-deficient mutant TBX5-K339R, which is not acetylated by KAT2A or KAT2B^17^. Promoter-reporter assays showed that the TBX5-K339R mutant still retained a significant amount of transcriptional activity on *MYH6* promoter (Fig. 3A). Co-transfection of TBX5-K339R along with HDAC4 or HDAC5 significantly reduced the transcriptional activity of TBX5-K339R (P < 0.001), whereas HDAC6, which does not interact with TBX5, did not have any effect on TBX5-K339R activity (Fig. 3A).

Next, we checked whether TBX5-K339R retained HDAC5 binding activity. To address this we performed pull-down assays, and the results indicate that the mutant protein retained wild-type binding activity (Fig. 3B). Overall, these experiments suggest that in addition to direct TBX5 deacetylation and subsequent cytoplasmic relocalization, HDAC4 and HDAC5 can also exert their effect on TBX5 through structural modification of chromatin via their promoter recruitment.

### HDAC4 inhibits TBX5-MEF2C complex formation

**Figure 4:**
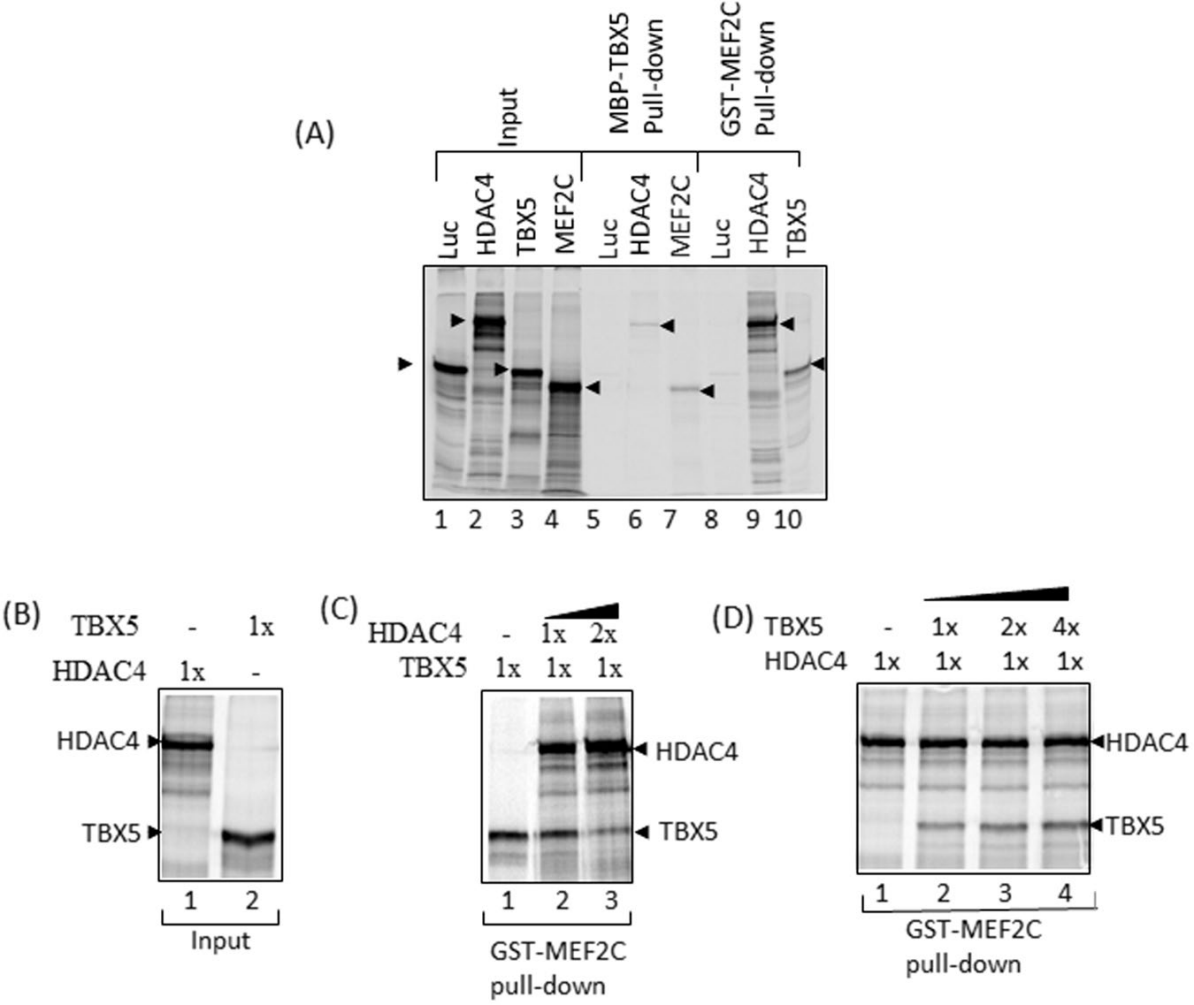
HDAC4 inhibits TBX5-MEF2C complex formation. (**A**) *In vitro* pulldown assay showing the interaction of TBX5 with HDAC4 and MEF2C (lanes 6 and 7)), and the interaction of MEF2C with HDAC4 and TBX5 (lanes 9 and 10). HDAC4 presents higher affinity for MEF2C than TBX5 does. Control protein luciferase (Luc) does not interact with TBX5 (lane 5) or MEF2C (lane 8). (**B**–**D**) Additional pull-down experiments indicate that increasing concentrations of HDAC4 (Fig **C**; lane 2 and 3) inhibit the TBX5-MEF2C complex formation. Increasing concentrations of TBX5 fail to compete out HDAC4 for MEF2C binding (Fig **D**; lane 2, 3 and 4).

Class II histone deacetylases HDAC4 and 5 associate with MEF2 and suppress MEF2-specific transcription, thereby regulating cardiac hypertrophy. TBX5 is an important partner of MEF2C and their functional cooperation is essential for heart development. Furthermore, our domain mapping studies also suggested that TBX5 interacts with the MADS-box of MEF2C, a region shared by HDAC4 and 5^18^. In view of these findings, we examined whether HDAC4/5 play a regulatory role in the TBX5 and MEF2C interaction by conducting i*n vitro* pull-down assays. In the first set of experiments, we incubated radiolabelled HDAC4 or MEF2C proteins with MBP-TBX5 protein, and in the second set of experiments radiolabelled HDAC4 and TBX5 were incubated with GST-MEF2C. Radiolabelled luciferase control protein was also included in the assay. Figure 4A shows the specific interaction of TBX5 with HDAC4 and MEF2C (lanes 6 and 7), and the interaction of MEF2C with TBX5 and HDAC4 (lanes 9 and 10). A relatively weak interaction was observed between HDAC4 and TBX5 whereas the interaction observed between HDAC4 and MEF2C was stronger. To analyse the effect of HDAC4 on TBX5-MEF2C complex formation, additional pull-down experiments were carried out. We found that by increasing the concentration of HDAC4, the formation of the TBX5-MEF2C complex was reduced, suggesting that HDAC4 displaced TBX5 from the MEF2C-TBX5 complex (Fig. 4C). However, when the concentration of TBX5 was increased there was no change in MEF2C-HDAC4 complex formation, suggesting that TBX5 was unable to displace HDAC4 from the MEF2C-HDAC4 complex (Fig. 4D).

Overall, these results suggest that HDAC4 inhibits the TBX5-MEF2C complex formation by competing out TBX5.

### HDAC4 inhibits MEF2C-TBX5 complex formation on MYH6-promoter target DNA and represses transcription

**Figure 5:**
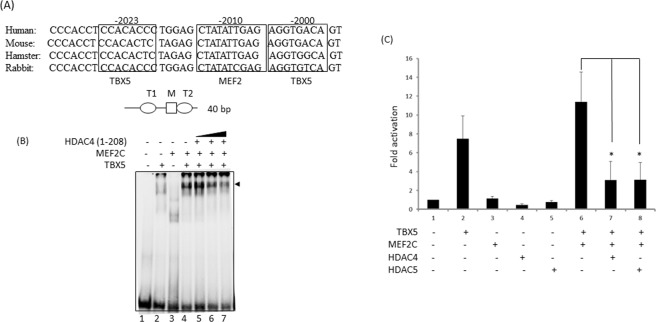
HDAC4 inhibits MEF2C-TBX5 complex formation on MYH6-promoter target DNA and inhibits its transcriptional activity. (**A**) 40 bp *MYH6* promoter sequence showing conserved TBX5 and MEF2C binding sites that was used for EMSA studies. (**B**) Electrophoretic mobility shift assay (EMSA) showing the formation of TBX5-MEF2C complex on the MYH6-promoter target DNA (lane 4, black arrowhead). Increasing concentrations of HDAC4 reduced such complex formation (lanes 5–7). (**C**) Luciferase-reporter assay on the MYH6 promoter indicates that HDAC4 and HDAC5 repress the TBX5-MEF2C transcriptional activity. Results are mean from three individual experiments. Error bars represent SD, *P < 0.02.

TBX5 and MEF2C form a complex on a *MYH6* promoter fragment^18^. Since HDAC4 competes with and prevents TBX5-MEF2C association, we investigated the impact of HDAC4 on TBX5-MEF2C complex formation on DNA target. Gel mobility shift assays revealed that TBX5 and MEF2C form a strong complex on the MYH6-promoter fragment (T-M-T) (Fig. 5A,B). When HDAC4 was included in the binding assays, TBX5-MEF2C complex formation on the target was hindered, suggesting that HDAC4 inhibited the TBX5-MEF2C complex formation on its target DNA (Fig. 5B). This finding was consistent with our results from competition assays.

The TBX5 and MEF2C functional interaction plays a major role in cardiac gene transcription. The interaction of HDACs with TBX5 prompted us to analyse the effect of HDAC4 and 5 on TBX5-MEF2C-mediated transcription. As shown in Fig. 5C, transfection of TBX5 and MEF2C together led to a significantly higher activation level of the MYH6 promoter than transfection of TBX5 alone. When either HDAC4 or 5 were co-transfected along with TBX5 and MEF2C, the functional cooperation between them was significantly reduced (P < 0.004) as shown by the decreased fold-activation of the MYH6 promoter.

Thus, these experiments suggest that HDAC4 and HDAC5 strongly inhibit TBX5-MEF2C complex formation on their promoter target DNA, inhibiting their functional cooperativity.

### PRKD1 relieves HDAC4 and 5-mediated repression on TBX5

**Figure 6:**
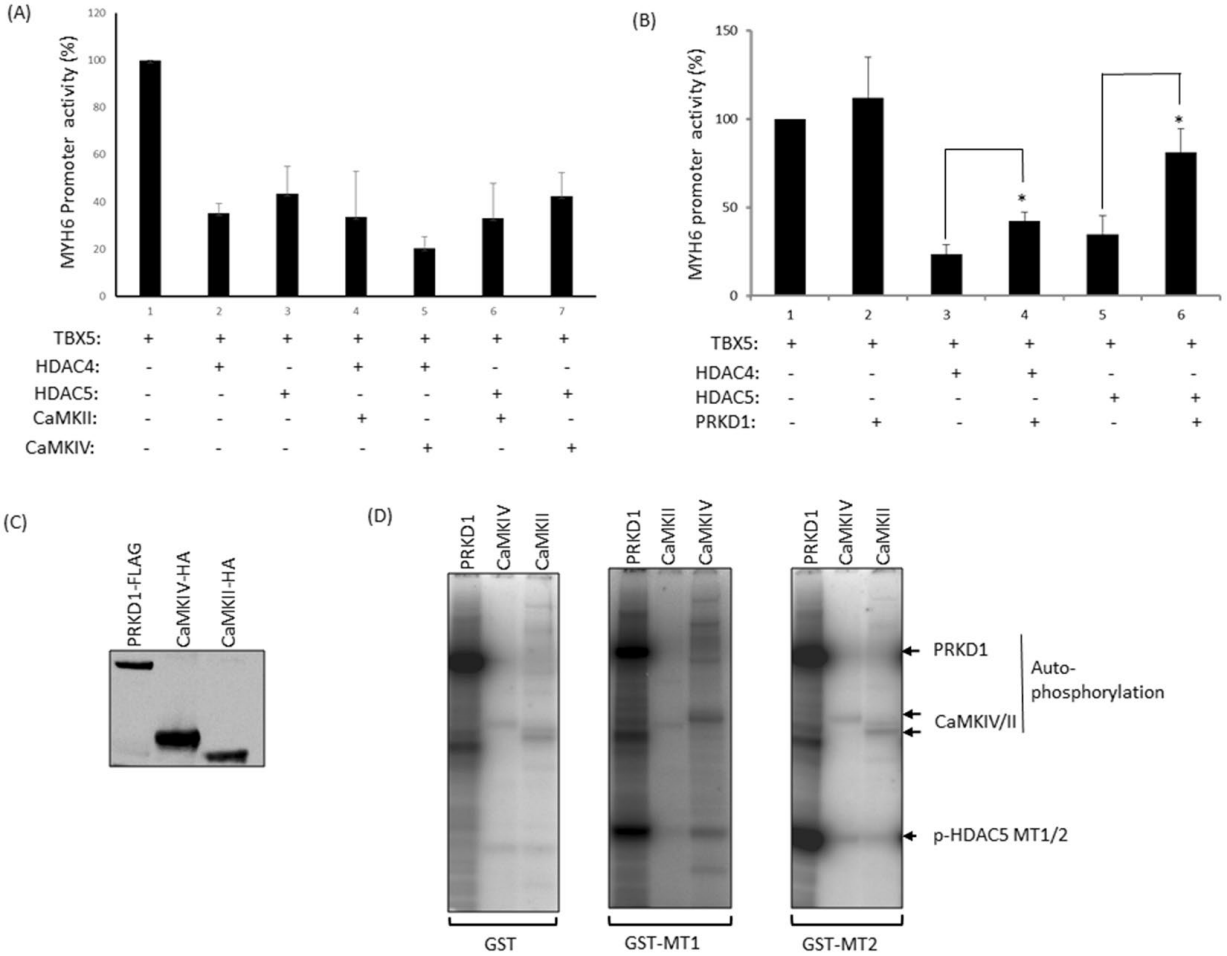
PRKD1 relieves HDAC4 and 5-mediated repression of TBX5. (**A**) Luciferase-reporter assay showing that CaMKII and CaMKIV can not relieve HDAC4/5-mediated repression of TBX5 on a MYH6 promoter fragment. (**B**) Luciferase-reporter assay shows that PRKD1 does relieve the HDAC4 and HDAC5-mediated repression of TBX5 on the MYH6 promoter. Results are means from three individual experiments, error bars represent SD, *p < 0.05. (**C**) Western blot showing the level of PRKD1, CaMKII and CaMKIV proteins used for phosphorylation assays. Full-length blot is shown Supplementary Fig. S4. (**D**) *In vitro* phosphorylation activity of PRKD1, CaMKII and CaMKIV on HDAC5 phosphorylation motifs MT1 (S259) and MT2 (S498) (bottom arrowhead). Both motifs were strongly phosphorylated by PRKD1, whereas phosphorylation by CaMKII/CaMKIV was very much weaker.

HDAC4 and HDAC5 are signal-responsive repressors of cardiac hypertrophy. Signal-mediated kinases CaMKII and IV and serine-threonine protein kinase D1 (PRKD1) phosphorylate class IIa HDACs. This post-translational modification promotes their nuclear export, relieving repression of MEF-dependent gene transcription^10,12,19^. Similar to MEF2, HDAC4 and 5 also associate with TBX5 and repress TBX5-mediated transcription. To investigate whether CaMKII and IV play any roles in TBX5-dependent gene transcription we performed reporter assays. As shown in Fig. 6A, neither CaMKII nor CaMKIV showed any effect on TBX5-mediated activity. When either CaMKII or CaMKIV were co-transfected with TBX5 and HDAC4 or TBX5 and HDAC5, neither kinase successfully relieved the repression imposed by HDAC4 and 5. However, in similar experiments, co-transfection of PRKD1 partially relieved both HDAC4 and HDAC5-mediated repression on TBX5 (Fig. 6B, P < 0.05). Western blot analysis suggests Tbx5, Hdac5, Hdac4 and Prkd1 are co-expressed in the mouse embryonic heart (Fig. S6).

To further substantiate the distinct mechanism between these kinases, we investigated their *in vitro* phosphorylation activity on HDAC5. HDAC5 is phosphorylated at S259 and S498 by these kinases. We generated two GST-fusion proteins corresponding to the motif 1 (GST-MT1) and motif 2 (GST-MT2) of HDAC5 containing the Ser259 and Ser498 respectively. These two motifs are highly conserved among class II HDACs. PRKD1-FLAG and CaMKII/IV-HA fusion proteins were generated in Cos7 cells (Fig. 6C). Kinase assays revealed that both Ser259 and Ser498 were strongly phosphorylated by PRKD1, whereas a very weak phosphorylation of these two serine residues was observed with both CaMKII and CaMKIV (Fig. 6D). A loading control for the kinase assay gel is shown in Fig. S5. The very weak phosphorylation of HDAC5 by CAMKII/IV correlates with their inability to de-repress TBX5 in the reporter assays.

Overall, these experiments suggest that PRKD1 relieves repression of TBX5 possibly due to nuclear export of HDAC4/5 upon phosphorylation.

## Discussion

Building the heart is a complex process governed by a network of molecules such as transcription factors, cofactors, signalling molecules and structural proteins^20,21^. As part of this complex network, TBX5 directly interacts and functionally cooperates with other cardiac transcription factors, most notably GATA4^22^, MEF2C^18^ and MYOCD^23^ among others. The functional cooperation between these factors is crucial for proper heart development, and mutations that disrupt such association often lead to congenital heart diseases (CHDs). TBX5 activity can also be modulated at a post-translational level, as our recent studies suggest that acetylation of TBX5 enhances its transcriptional potential^17^, whereas others have shown the importance of sumoylation^24^.

HDACs are modulators of heart development and growth, and their association with MEF2 transcription factors are central to the control of myogenesis and cardiac hypertrophy^10,25^. Our pull-down assays suggest that TBX5 associates with both class I (HDAC1, 2 and 3) and class II HDACs (HDAC4 and 5). Association of Tbx5 and Hdac2 in the Nucleosome Remodeling and Deacetylase (NuRD) complex has been reported previously but Hdac4 and 5 were not detected in that complex^20^. The association of TBX5 with HDAC4/5 is particularly interesting, not only because they are abundantly expressed in the heart^11^, but also due to their roles in cardiac hypertrophy^10^. The association of TBX5 with HDAC4/5 resulted in repression of TBX5-mediated transcription of cardiac gene *MYH6*. In addition, HDAC4/5 deacetylate TBX5 leading to its partial redistribution into the cytoplasm. Interestingly, the non-acetyl version TBX5-K339R also retained HDAC5 binding activity and was repressed by both HDAC4/5 in reporter assays suggesting that a chromatin restructuring mechanism does play a significant role in the transcriptional regulation of TBX5. Taking all these facts into account, we suggest that the repression of TBX5 activity by HDAC4/5 could be attributed to two factors; 1) deacetylation of TBX5 that promotes its nuclear export which effectively reduce the nuclear pool and 2) alter chromatin structure due to TBX5-mediated promoter recruitment of HDAC4/5.

Functional cooperation between TBX5 and MEF2C is essential for early heart development^18^. In pull-down assays we found that HDAC4 associates with both TBX5 and MEF2C, however the association between HDAC4 and MEF2C was stronger. In competition assays, HDAC4 displaced TBX5 from the TBX5-MEF2C complex, whereas TBX5 was unable to compete with HDAC4 for MEC2C binding. In addition, HDAC4 inhibited TBX5-MEF2C complex formation on the target DNA and supressed their functional cooperation on a *MYH6* promoter. TBX5 interacts with the MADS-box region (1–60 Aa) of MEF2C^18^ and HDAC4 interacts overlapping MADS-MEF2 region (39–72 Aa)^25^. Since the MEF2C interacting region for TBX5 and HDAC4 overlap significantly, it is likely they compete for MEF2C binding. In addition to cytoplasmic re-localization and alter chromatin structure, our results also suggest that HDAC4/5 disrupt the association and functional cooperation between TBX5 and MEF2C, thus ensuring maximum TBX5 repression.

Class II histone deacetylases are signal responsive repressors of cardiac hypertrophy^10^. Signal-mediated protein kinases such as CaMKII/IV and protein kinase A/D (PRKA/D) phosphorylate specific serine residues in HDAC4/5 that guide them into the cytoplasm and relieve repression of MEF2-mediated gene transcription^10,26^. Our studies suggest that it is PRKD1, and not CaMKII/IV, that partially relieves HDAC4/5-mediated repression of TBX5. The PRKC/PRKD signalling pathway promotes agonist-dependent cardiac hypertrophy through phosphorylation of HDAC5 and nuclear export^15^. PRKD-dependent phosphorylation of HDAC5 occurs at Ser 259 and Ser 498 and is critical for nuclear export^27^. Our studies suggest that PRKD1 strongly phosphorylates Ser259 and Ser498 on HDAC5, and that could be linked to de-repression of TBX5 target genes. It is also possible that PRKD1 could directly phosphorylate TBX5 and potentiates its transcriptional activity. CaMKII and CaMKIV, which do not de-repress TBX5, showed very weak phosphorylation activity in our assay conditions.

TBX5 plays a key role in heart development and disorder. Many key components that interact and modulate the function of TBX5 have been identified, thus helping us to understand the molecular mechanisms of heart development and disease. In this study we revealed a novel TBX5-HDAC4/5-PRKD1 gene regulatory pathway, which might be important for several aspects of heart development, and which warrants further investigation.

## Materials and Methods

### Generation of plasmids

The plasmid pcDNA-HDAC1-FLAG was a generous gift from Marian A. Martinez-Balbas (Barcelona, Spain). Plasmids pME18S-FLAG-Hdac2 and pcEP4F-FLAG-HDAC3 were a gift from Dr Y. E. Chin (Rhode Island Hospital). Hdac2-FLAG was subcloned into the EcoRI and XhoI sites of pcDNA3.1. The plasmid pcDNA-HDAC4-FLAG was a generous gift from Dr S.L Schreiber (Cambridge, USA). Plasmids pcDNA-HDAC5-FLAG and pcDNA-HDAC6-FLAG were generated from IMAGE clones 6043491 and 2984860 respectively (Source Biosciences). Plasmids pcDNA-HA-CaMKIIδ and pcDNA-HA-CaMKIV were made from IMAGE clones BC032784 and BC025687 respectively (Source Biosciences). The plasmid pcDNA-PRKD1 was generated by cloning *PRKD1* cDNA into the EcoR1 and XhoI sites in pcDNA3.1. The original clone was obtained from Source Biosciences (IMAGE: 100063951, Ac no: BC160015). We also cloned two DNA fragments into pGEX4T-1 encoding the phosphorylation motifs of HDAC5: MT1 and MT2 (corresponding to the amino acid residues 254 to 264 and 493 to 503 respectively) to generate fusion proteins GST-MT1 and GST-MT2.

### Cell transfection and reporter assay

This was conducted as described previously^18^. Rat cardiomyoblast cell line H9c2 and Cos7 cells were maintained in Dulbecco’s modified Eagle’s medium (DMEM) supplemented with 10% fetal calf serum. For reporter assays, all transfections were performed in 6 wells plates using Polyfect reagent (Qiagen) according to the manufacturer’s protocol. Cells in each well received 1.5 μg of reporter plasmid, 1.0 μg of expression plasmids pcDNA-TBX5 and 0.5 µg of pcDNA-HDAC1, pcDNA-Hdac2, pcDNA-HDAC3, pcDNA-HDAC4, pcDNA-HDAC5 or pcDNA-HDAC6. For TBX5 and MEF2C experiments we also added 0.25 μg pcDNA-MEF2C. For CaMKII and CaMKIV experiments we co-transfected 0.5 µg of pcDNA-CaMKII or pcDNA-CaMKIV. To normalize the variation in transfection efficiency between the plates 4 ng of plasmid pRL-TK was used. The total amount of plasmid DNA in each well was adjusted to 3.0 or 3.5 μg with empty vector pcDNA3.1 as appropriate. After 24 h of transfection cells were harvested in lysis buffer and luciferase activity was measured using a Dual Luciferase assay kit (Promega). Each transfection experiment was carried out in duplicate and repeated for at least three times. Values shown in the histograms are means ± SD. Significance of analysis was performed by student t-test.

### *In vivo* pull-down assay and Western blot analysis

Cos7 cells were grown in 100 mm petri dishes in DMEM media. Cells were transfected with 6.0 µg of pcDNA-Myc-TBX5 and 4.0 µg of pcDNA-FLAG-HDAC plasmid DNAs using transfection reagent polyfect (Qiagen). In some experiments, we also included 4.0 µg of pcDNA-HA-CaMKII or pcDNA-HA-CaMKIV. Transfected cells were harvested after 48 h and lysed in lysis buffer (20 mM Tris (pH-7.6). 150 mM NaCl, 0.5% NP40, I mM PMSF, 1 mM aprotinin, 1 mM pepstatin and 1 mM leupeptin). Cells were left in lysis buffer for 1 h under ice, followed by brief sonication. The extracts were centrifuged at 12,000 g for 10 mins at 4 °C and stored at −80 °C until used.

Pull-down assays were carried out as described previously^17^. Cell extracts were incubated with 20–30 µl of washed Anti-FLAG M2-Affinity gel (Sigma) at 4 °C. For interaction studies, cell lysates were added to the affinity gel and incubated for 2 h or overnight in a shaker at 4 °C. To remove non-specific proteins, beads were washed 6 times in wash buffer (20 mM Tris (pH7.6), 150 mM NaCl, 0.5% NP40 and 0.5 mM EDTA). Bound proteins were specifically eluted from the beads with wash buffer (15.0 µl) containing 3X FLAG peptide (Sigma). Eluted proteins were fractionated on 10% Bis-Tris gels followed by Western blotting. FLAG-tagged, Myc-tagged and HA-tagged proteins were detected using monoclonal Anti-FLAG M2 antibody (Sigma), Anti-Myc antibody (Millipore) and Anti-HA antibody (Sigma) respectively.

Mouse heart extract was generated in 8 M urea buffer (20 mM Tris (pH-7.6, 150 mM NaCl, 8 M urea and protease inhibitors) from E13.5 embryonic hearts. Protein estimation was carried out using Quick start Bradford protein assay reagents (Bio-Rad). Western detection was carried out on 10 µg of extract with anti-TBX5 (Abnova), anti-HDAC5 (abcam), anti-HDAC4 (Santa Cruz), anti-PRKD1 (Sigma) and anti-GAPDH (Santa Cruz).

### Immunofluorescence and cell imaging

This was performed as described previously^17^. Cos7 cells were grown on coverslips in 6-well plates overnight and transfected with TBX5-V5 and HDAC4-FLAG/HDAC5-FLAG constructs using polyfect (Qiagen). Twenty-four hours post-transfection cells were washed in phosphate buffer saline (PBS, pH 7.4), fixed with 4% paraformaldehyde for 10 mins and washed in PBS three times, 5 minutes each. Cells were then permeabilized with 0.25% Triton X-100 in PBS for 5 minutes washed in PBS three times and incubated in 10% BSA in PBS for 1 hour at room temperature to supress non-specific binding of IgG. After blocking the cells were washed in PBS and incubated with mouse monoclonal antibody against V5 (ThermoFisher, R960, dilution 1:400) or with rabbit monoclonal antibody against FLAG (Sigma, F7425, dilution 1:500) in PBS containing 3% BSA for 2 hours at room temperature. Cells were washed three times in PBS each for 5 minutes and incubated in 3% BSA in PBS containing secondary antibody Alexa Fluor 488 goat anti-rabbit IgG and/or Alexa Fluor 594 goat anti-mouse (Life Technology, 2 µg/ml) for 45 minutes at room temperature. Then cells were washed three times in PBS each for 5 minutes and the washed coverslips were mounted on glass slides containing a small drop of mounting media containing DAPI (Vectashield). Laser scanning confocal microscopy was performed on LSM 880 F (Carl Zeiss, Oberkochen, Germany) using a 63 × 1.4 oil objective.

### *In vitro* coupled transcription/translation

*In vitro* synthesis of full-length and truncated TBX5 and MEF2C proteins was performed using coupled reticulocyte lysate (Promega) according to the manufacturer’s protocol and was described previously^18^. Briefly, 1.0–2.0μg of template DNA was incubated at 30 °C for 90 minutes in a 50μl reaction mixture containing ^35^S-methionine (Amersham). Following incubation protease inhibitors PMSF (1 mM), leupeptin (1μg/ml), pepstatin (1μg/ml) and aprotinin (1μg/ml) were added to protect samples from proteolysis. Proteins were visualized using SDS-PAGE followed by autoradiography.

### Protein expression, purification and kinase assays

FLAG-PRKD1, HA-CaMKII and HA-CaMKIV proteins were overexpressed in Cos7 cells by transfecting the cells with plasmid pcDNA-PRKD1 or pcDNA-CaMKII or CaMKIV and ployfect reagent (Qiagen). Cell extracts were generated from 48 h post transfected cells. Both FLAG and HA-tagged proteins were partially purified on anti-FLAG-Agarose and anti-HA-Agarose affinity gels (Sigma) respectively, aliquoted into tubes and stored at 4 °C until use. Glutathione S-transferase (GST), GST-MT1 and GST-MT2 proteins were purified on a GST-purification kit (GE healthcare Life Sciences). Protein concentration was determined by Quick start Bradford protein assay kit (Bio-Rad).

Kinase reactions were assembled in Eppendorf tubes containing beads immobilized with FLAG-PRKD1 or HA-CaMKII/IV proteins. In each tube we added 5 µl of 5X kinase buffer (150 mM Tris-HCl buffer (pH-7.5), 100 mM MgCl_2_, 1 µl of cold ATP (5 mM), 1 µl Ƴ^32^P ATP and 4 µl of GST proteins (4 µg)). For CaMKII/IV we included 1 mM CaCl2, 0.6 mM calmodulin in the kinase buffer. The final volume of the reaction mix was kept to 25 µl. Tubes were incubated at 30 °C for 45 mins. In each tube 6 µl of 4X LDS sample buffer (Invitrogen) and 1 µl of reducing agent (Invitrogen) were added and heated at 95 °C for 8 mins. Tubes were spun down and 20 µl of each sample was loaded on 10% Bis-Tris gel (Invitrogen) and electrophoresed for 1 h at constant voltage. The gel was washed in dH_2_O and stained with Simply blue stain (Invitrogen). The gel was then de-stained with dH_2_O, dried and subjected to autoradiography.

### *In vitro* pulldown assay

This was performed as described previously^18^. Full-length or truncated versions of TBX5 proteins were generated as MBP-fusions in bacteria following IPTG induction. These fusion proteins were partially purified from bacterial lysates on amylose resin. Their integrity was checked on PAGE by Coomassie blue staining of the gel. MBP-TBX5 fusion proteins were immobilized on amylose resin and GST-MEF2C was immobilized on glutathione sepharose beads for interaction studies. Equivalent amounts (2–5 μg) of immobilized MBP or GST fusion proteins were incubated with ^35^S-labelled full-length MEF2C or TBX5 (wild type or mutant) in binding buffer (50 mM Tris pH 7.4, 100 mM NaCl, 0.05% NP40, 1 mM DTT, 10% glycerol, 0.5 mM PMSF, 1μg/ml aprotinin, 1μg/ml pepstatin, 1μg/ml leupeptin, 0.05% BSA) for 2 hours at 4 °C. Beads were washed four times with binding buffer without BSA before the bound proteins were released from the beads by boiling with SDS sample buffer. Eluted proteins were subsequently resolved on SDS-PAGE followed by scanning on a Storm PhosphorImager (Amersham Bioscience), and band intensity was quantified using Image Quant software (Molecular Dynamics).

### Gel mobility shift assay

We performed electrophoretic mobility shift assays (EMSAs) as described previously^28^. A DNA fragment corresponding to bases –1491 to –1530 of the *MYH6* promoter containing two TBX5 sites flanking a (A/T)-rich site (T-M-T) was used as a probe. The probe was prepared by annealing the complementary oligonucleotide corresponding to oligonucleotide CAC CTC CAC ACC CTG GAG CTA TAT TGA GAG GTG ACA GTA AAC and end-labelling. All the reactions were carried out in 15–20 μl volume and incubated for 0.5 h at room temperature.

### De-acetylation of TBX5

Cos7 cells were transfected with 6.0 µg pcDNA-FLAG-TBX5, 2.0 µg pcDNA-HA-KAT2B and either with 2.0 µg pcDNA-FLAG-HDAC4 or pcDNA-FLAG-HDAC5. Cell extracts were generated in lysis buffer as stated above from 48 h post-transfected cells. FLAG-TBX5 protein was partially purified from the extract and fractionated on 10% bis-Tris gel (ThermoFisher Scientific) followed by Western blotting. The acetylation status of TBX5 was monitored with anti-Acetylated- Lysine antibody (Cell Signaling).

### Ethical statement

All the experiments were performed in accordance with relevant guidelines and regulations of the Animal Procedures Act under license number P375A76FE (Nottingham), issued 2018-08-22, and granted by The Home Office. All methods were carried out in accordance with relevant guidelines and regulations

### Statistical analysis

All the data shown here are means ± standard deviation (SD). The significance of differences between the groups was checked by student’s t test. The P value less than 0.05 were considered as significant.

## Supplementary information

Supplementary information
