# Supplementary material for: HDAC4 and 5 repression of TBX5 is relieved by protein kinase D1
Source: Sci Rep. 2019 Nov 29;9:17992. doi: 10.1038/s41598-019-54312-w (PMC6884511; doi:10.1038/s41598-019-54312-w)

**HDAC4 and 5 repression of TBX5 is relieved by protein kinase D1**

Tushar K Ghosh, José J Aparicio-Sánchez, Sarah Buxton and J David Brook^1^

School of Life Sciences, Queen's Medical Centre, University of Nottingham, Nottingham NG7 2UH, UK.

^1^Corresponding author: david.brook@nottingham.ac.uk

**Legends for Supplementary Figures:**

**Supplementary Figure S1**: A) Full-length blots that are used for generating the Fig 1A are shown. Lane 1 –MagicMark (Invitrogen); lane 2-TBX5-MYC, lane 3-TBX5-MYC + HDAC1-FLAG, lane 4-TBX5-MYC + HDAC2-FLAG; lane 5- TBX5-MYC + HDAC3-FLAG, lane 6- TBX5-MYC + HDAC4-FLAG, Lane 7- TBX5-MYC + HDAC5-FLAG, lane 8- TBX5-MYC + HDAC6-FLAG. The boxed areas are shown in Fig 1A.

**Supplementary Figure S2**. Complete Western blot for Fig 2A and 2B. The Boxed regions are shown in Fig 2A and 2B. Blots A and B are for TBX5 deacetylation by HDAC4. Lane 1 -MagicMark (Invitrogen), lane 2-TBX5-MYC, lane 3-TBX5-MYC + HDAC4-FLAG. Blot B is re-probing of the blot A. Blots C and D are for TBX5 deacetylation by HDAC5. Lane 1- MagicMark, lane 2- TBX5-MYC, lane 3- TBX5-MYC +HDAC5-FLAG. Blot D is re-probing of blot C.

**Supplementary Figure S3**. Full-length Western blots shown in Fig 3B. The Boxed regions are shown in Fig 3B. TBX5 is 60kDa, HDAC5 is 120kDa. Lane 1-MagicMark (Invitrogen), lane3-TBX5-MYC, lane4-HDAC5-FLAG + TBX5-MYC, lane 5-TBX5K339R –MYC. Lane 6-TBX5K339R-MYC +HDAC5-FLAG, the blot C is re-probing of the blot A. IB: Immunoblot.

**Supplementary Figure S4**. Full-length Western blot shown in Fig. 6C. The boxed region is used to produce the Fig. 6C. Lane 1-PRKD1-FLAg, lane 2-caMKIV-HA and lane 3-CaMKII-HA. The blot was probed with both anti-FLAG and anti-HA antibodies.

**Supplementary Figure S5**. Coomassie blue stained gels showing the level of PRKD1, CaMKIV and CaMKII used for the kinase assays. The same gels were dried and subjected to autoradiography. Asterisk denotes GST protein band from the GST-MT1/GST-MT2 fusion proteins.

**Supplementary Figure S6**. Western blot analysis showing the co-expression of Hdac5 (A), Hdac4 (B), Tbx5 (C) and Prkd1 (D) in the mouse embryonic heart tissue (E13.5). Gapdh is used as loading control (at the bottom). Asterisk denote a non-specific/processed product. Lane 1-MagicMark Western protein standard (Invitrogen). Lane 2 –heart extract.


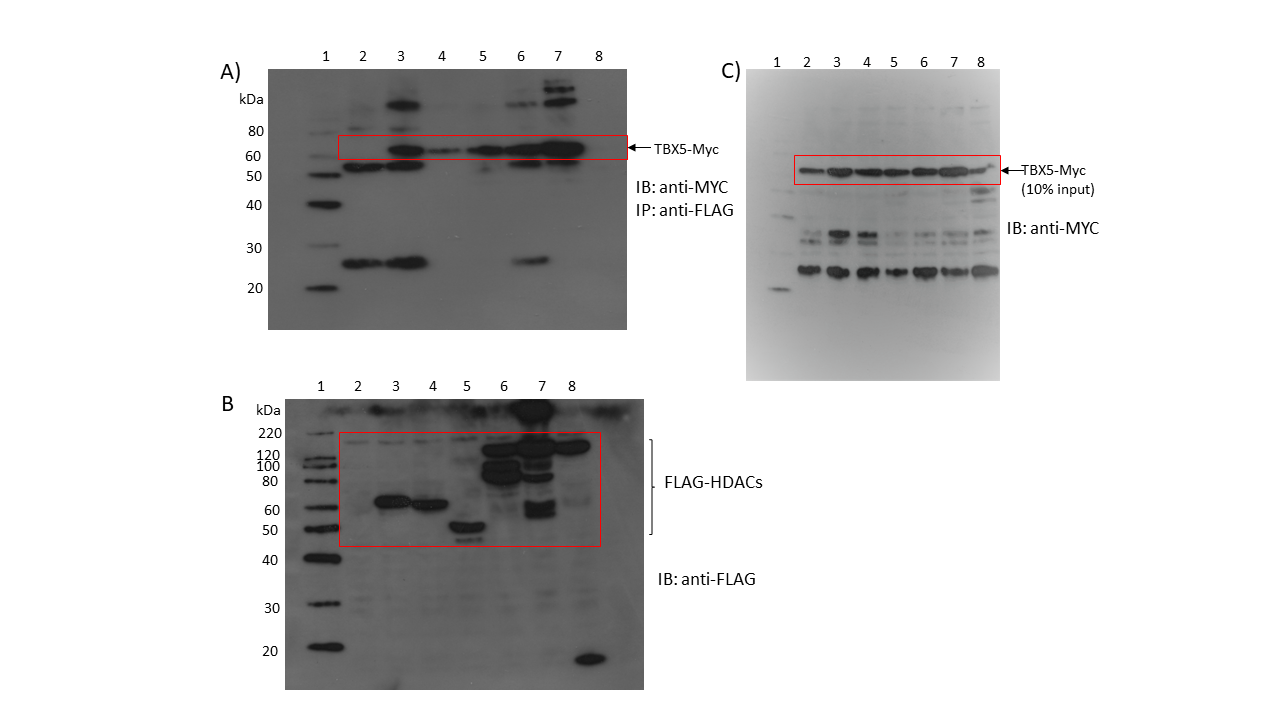


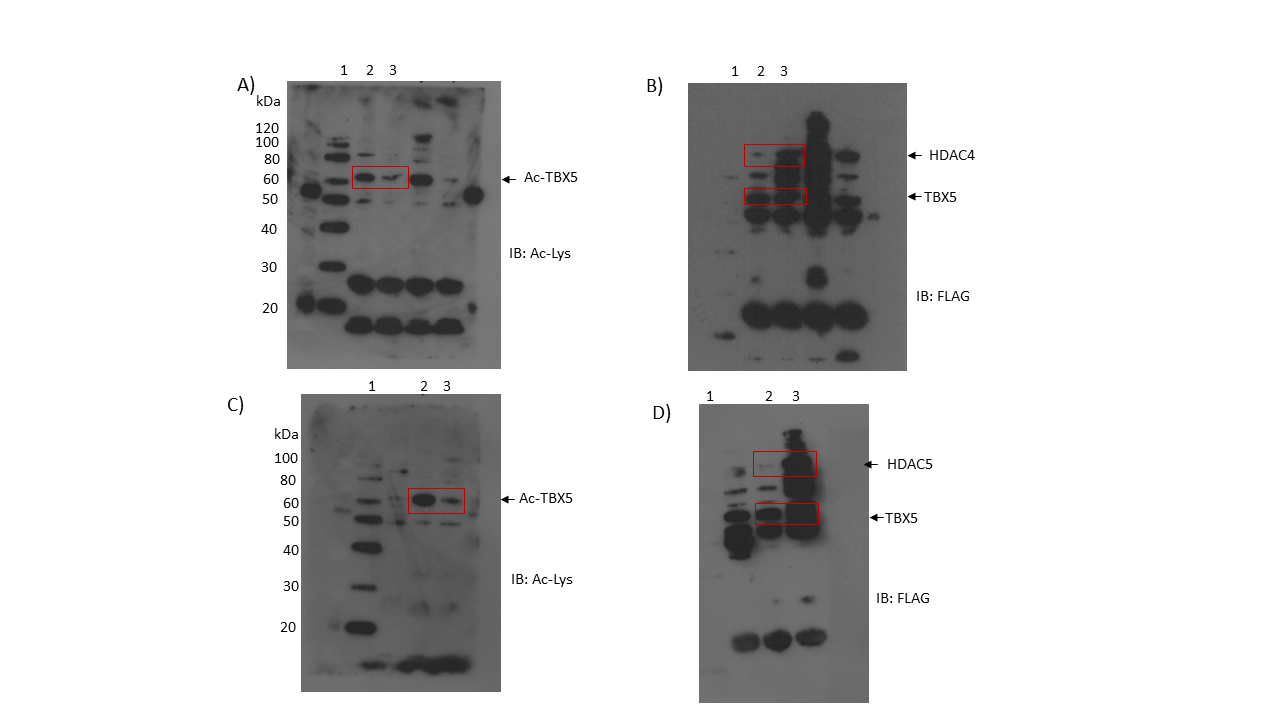


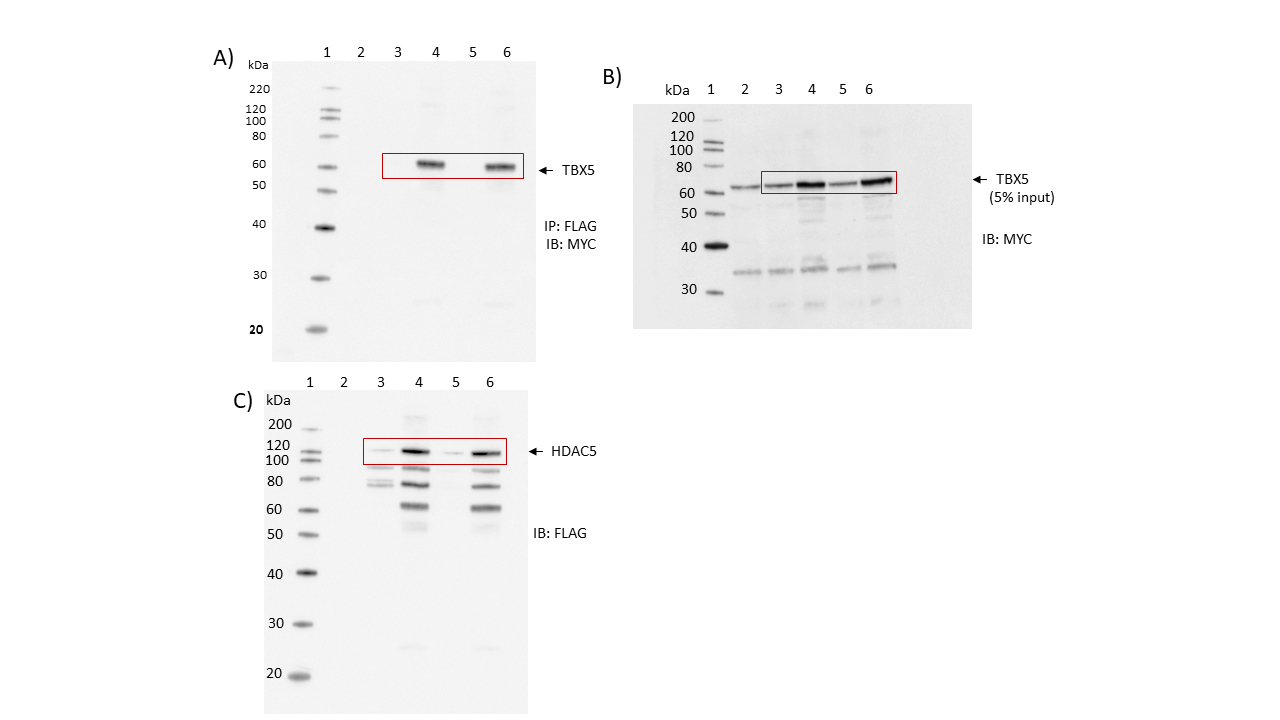


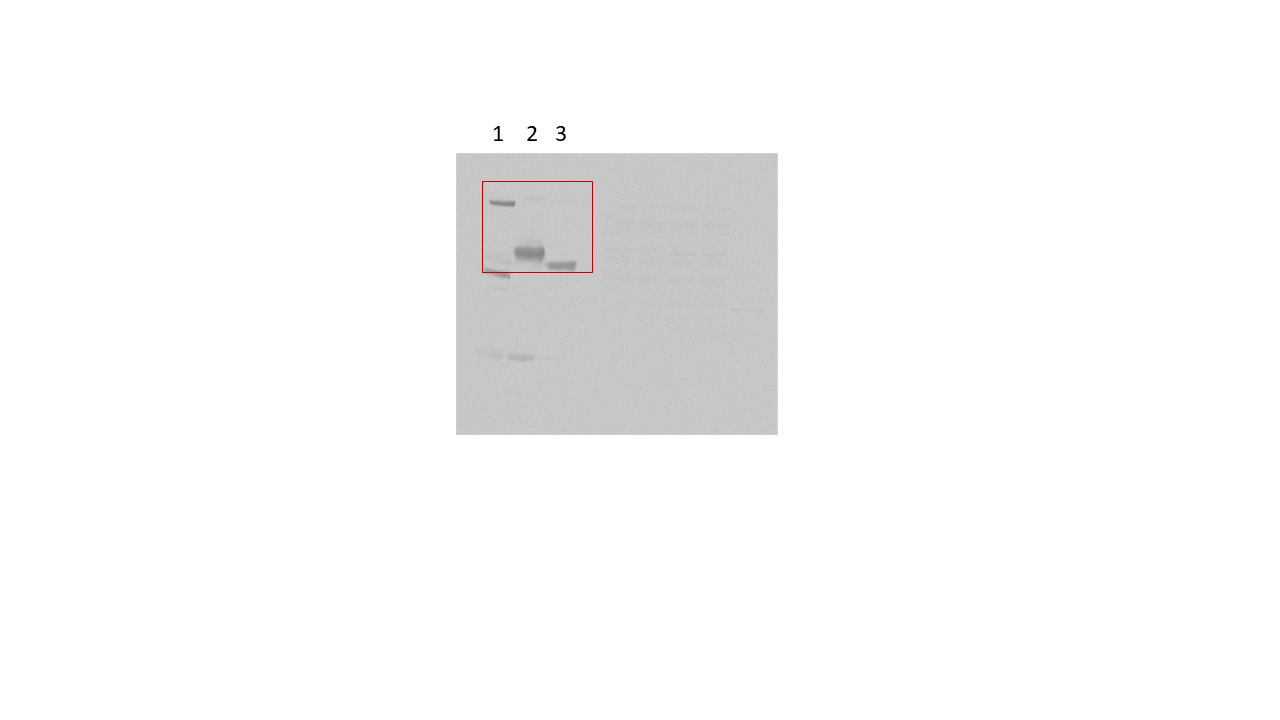


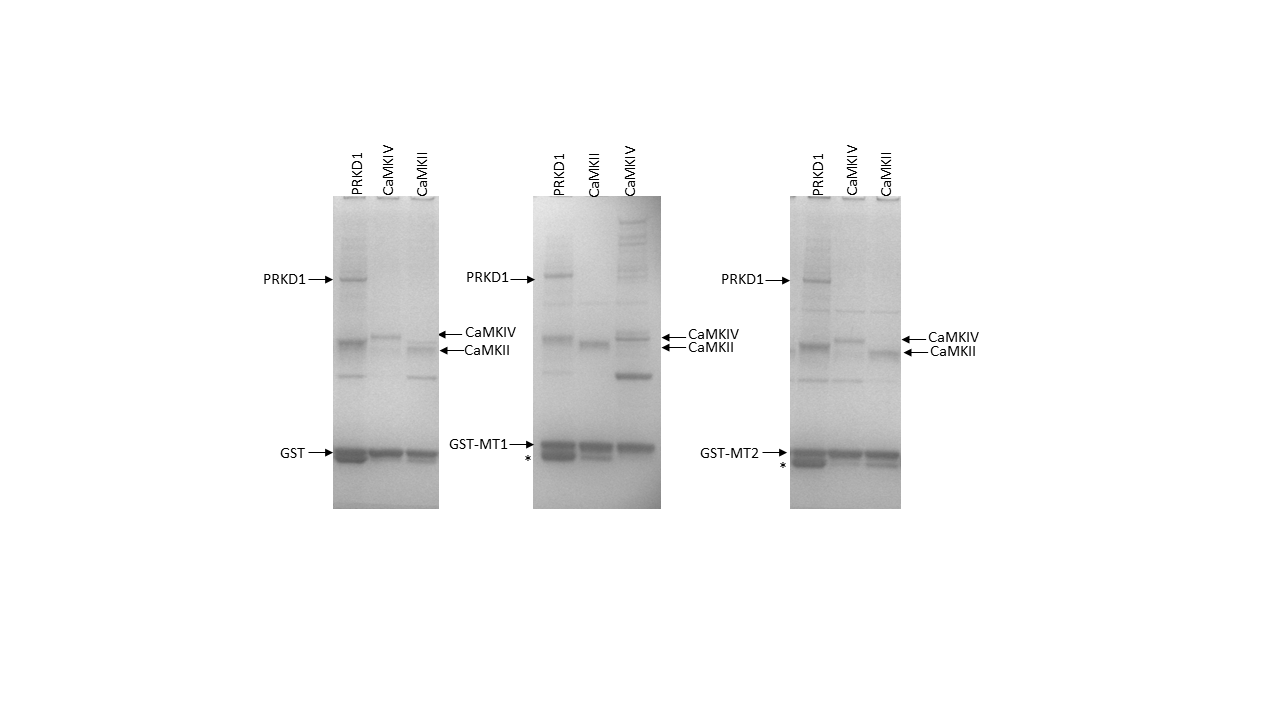


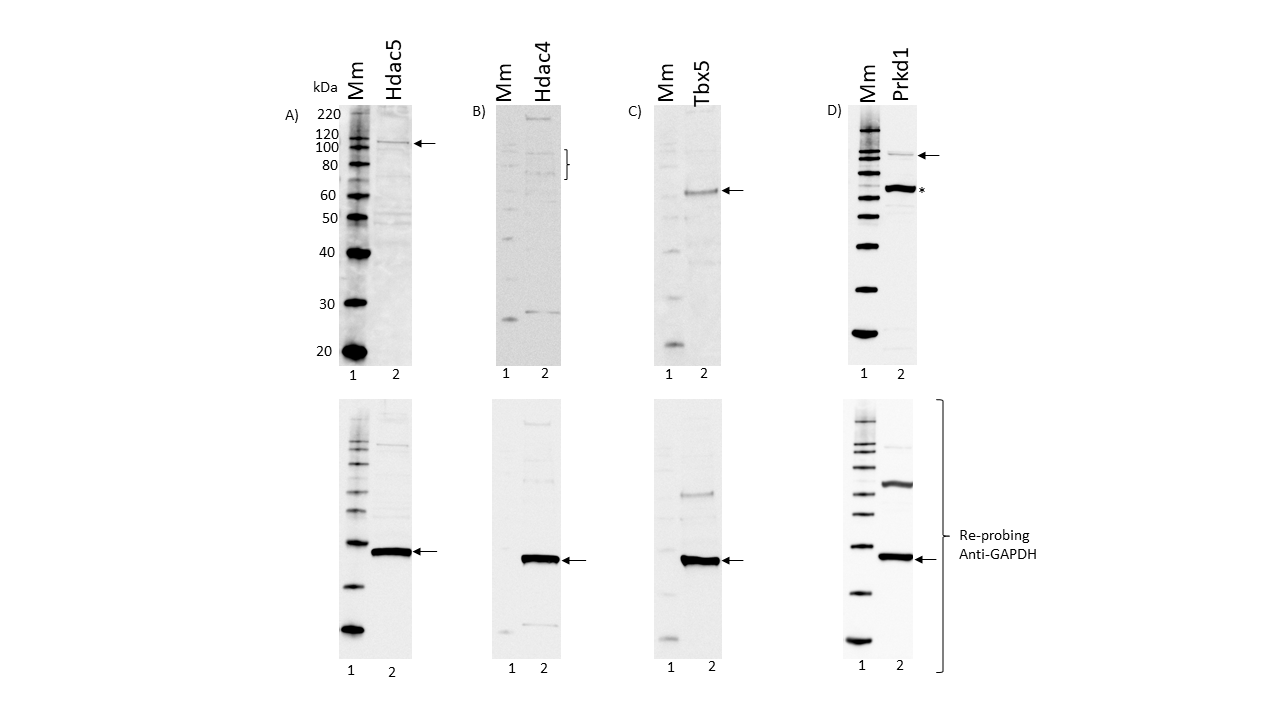

Supplement: Supplementary file 1 — Supplementary information [file 41598_2019_54312_MOESM1_ESM.docx]
